# The Spirit Bottle

**Published:** 1887-12-17

**Authors:** 


### The Doctor's Pulpit.

### THE SPIRIT BOTTLE.

A Medical Practitioner was not long ago called to see a poor woman who was said to be dangerously ill. He found her suffering from "hobnail" liver, a condition often brought about by the drinking of bad spirits, but especially of gin. Her body and legs were swollen and dropsical, her face was puffy, the whites of her eyes were muddy and yellow-looking. She was dull and stupid, and constantly asked for gin, which had come to be almost the only thing she would willingly take. The doctor, who was young, and comparatively new to London practice, felt a good deal shocked. He saw the woman was within a measurable distance of her death, and that she was in such a stupefied condition as to render it very improbable she could make any effort on her own behalf. Nevertheless, he resolved to try her. Tapping her on the forehead vigorously, he succeeded in bringing her mind momentarily to a state of attention. "You are very ill," said he.

"I am," she replied.

"You are dangerously bad."

"Yes," she answered again.

"Do you know that gin is killing you?"

"Yes."

"You will certainly die if you don't give it up."

"Then I'll die, for I can't and I won't give it up."

She did die in about a week. No doubt at the time when the doctor saw her it was impossible for her to leave off the spirit voluntarily, and even if she had done so it would have been impossible for her to recover. Nevertheless, at an earlier stage of the disease the giving up of her bad habits would have resulted in her restoration to health. She undoubtedly killed herself. She was about forty years of age.

Within a few months the same medical man had on his visiting list a young fellow of about thirty-three, the only son of his mother, who was a widow. He was suffering from the same disease as the woman, but at an earlier stage. The cause was the same—drunkenness; and the young man's drink consisted largely of spirit—coarse, common, bad spirit. He, however, drank beer as well, or anything else he could lay hands on. The doctor, as in the former case, made every possible effort to persuade the young man to better habits. He was successful for a time, and there was a decided improvement in the health as well as in the habits of his patient. He relapsed, however, in five or six weeks, and then drank more heavily than before. In two months from his relapse he also was dead.

Within two years of that time the doctor had signed death certificates for five other persons, the cause of death in each case being a disease, or a complication of diseases, resulting entirely from alcoholic habits. Of those five persons four were women—educated women—and one of them a lady of high social position only thirty-four years of age.

Was not such a three years' experience as that an ample reason for the conversion of the young medical man to the principles and practice of total abstinence? It was if he had been a man accustomed to act on impulse. But should a man who has come to years of discretion allow himself to act on impulse alone? No doubt in many cases impulses may and ought to be followed. But these are usually cases in which immediate action is demanded, and in which the right course is as plainly obvious as the necessity for prompt action. For example, if we were to see a grown-up man illtreating a small and helpless child, impulse would suggest the knocking of the man down if nothing else were sufficient; and such a course of action would probably be productive of the best results, not only to the child but also to the ruffian and his castigator. But if the same child fell overboard in mid-ocean and her father, who could not swim, should follow his immediate impulse and jump after her, he would clearly be doing wrong. There would then be two helpless people in the water instead of one, and the drowning child's chances of rescue would be diminished by one-half, in consequence of the very natural but rash and wrong action of her parent, acting on his first impulse.

Clearly then there are some courses which impulse would prompt a man to follow which would be quite right, whilst there are others which impulse would prompt him to follow which would be quite wrong. The former are generally courses where immediate action is the only action possible, and where the right course is quite obvious. The latter are actions which do not necessarily come under this category.

It must be admitted that whatever be the dreadful array of facts which can be set in serried ranks before a man to induce him to become a total abstainer they are not of a character to rightly demand from him an immediate answer in the affirmative. An eloquent temperance lecturer may take a not unnatural pride and pleasure in a large number of "sudden conversions." Nevertheless, total abstinence, like religion, is one of those questions which demand to be settled slowly, calmly, and with the best reasoning powers a man possesses. It should be looked at on all sides and from every possible standpoint, and the conclusion arrived at should be the result of mature deliberation.

The taking of alcoholic drinks or the abstaining from them have risen to be social questions of a very high order of importance. He who undertakes to speak or write about them should be impressed with a due sense of responsibility. Flippancy and claptrap must both be ruled out of the field. In asking advice from a medical man on such points, the questioner would naturally put forward inquiries bearing on the "health" aspect of the case. Is alcohol a food? Is it a medicine? Does it do any good to the system in any way, either as a food or as a medicine? Is the good, if any, of such a character as alcohol alone is capable of imparting? Can no efficient substitute be found for it? Is the good such as to counterbalance the injurious effects of all kinds which certain people attribute to alcohol?

These are the kind of questions which men ask of the medical profession, and to which, it seems to us, they have a right to demand definite answers. Definite answers are given by leading medical men to these questions. Unhappily they are not all of the same character. Some doctors say alcohol is a food; others say it is not. Some say it is such a stimulant that no substitute equally valuable has been found. Others say that there are stimulants available which are quite equal to, or better than alcohol. That is really an unhappy, as well as an unsatisfactory, state of things; but it is quite true; and it is as well-known as true.

We hold that in all cases the most perfect candour is not only morally right, but the very best course to pursue for all concerned.

In offering a contribution on the question we lay no claim to infallibility. We do, however, wish it to be understood that we have honestly striven to look at the subject from every possible point of view, and that we give our judgment with a full sense of responsibility. Is alcohol a food? If we desired to make a show of scientific learning, we should say that we must begin by defining our terms; and we should therefore define what we conceive a "food" to be. Such a course might mystify the reader, but would probably add little to his useful knowledge. We prefer, therefore, to answer as plainly and directly as possible; and inasmuch as we claim no infallibility, our readers must attach their own value to the answers. Is alcohol a food? To some people it appears to be decidedly a food. So far as observation can make out it seems both to fatten and strengthen the body. Many persons will traverse these statements, and quote high medical opinion. To these it is answered that no opinion is of value if it is opposed to ascertained facts. The facts are as open to us as to any other medical or non-medical person; and the facts seem to us to show conclusively that alcohol both fattens and strengthens some persons, and that therefore it is and must be a true and proper food.

Alcohol is admittedly a stimulant. The medical profession is constantly asked: Is it the best of all stimulants? Is it a stimulant of such a nature as that no other known to science can on every occasion be substituted for it? Must we not sometimes, in order to do the very best we can for our patients, order them an alcoholic stimulant? Here again, it seems to us the answer must be, on the whole, in favour of alcohol. In many cases alcoholic stimulants are the best we can order; in some cases they are absolutely necessary; in some, a medical man would fail of his duty, and might be said to be guilty of his patient's death, if he did not prescribe alcohol.

These judgments, given with the fullest sense of responsibility, seem to us to express the convictions of the majority of thoughtful, cultured, and experienced medical practitioners. More dogmatic statements might be made, and sometimes are; but in this case we consider that the more dogmatic a man is on either side in the controversy, the less trustworthy he is as a scientific and practical guide.

Some persons may run away from the perusal of this paper and indulge themselves in the use of alcohol without stint or limit, thinking that they have medical justification for it. Such persons will be excessively foolish, for they will have entirely failed to appreciate the significance of what has been said. A salt bath is often a good thing for a man, but is that a reason why he should live in the sea all the year round? Castor oil is occasionally useful, and may sometimes be the very best thing that can be taken. Would that justify a person in taking castor oil at every meal of the day, and sipping two or three glasses between times? People should read with sense and intelligence. Those who are honest will do so; those who are determined to do only as they please will find justification anywhere. The general convictions of the present writer on the subject of alcohol are these: that it is a food; that for some people it may sometimes be a necessary food; that it is a valuable stimulant; that sometimes it is an absolutely essential stimulant. But although these things are so, yet as a rule the man or the woman who can do without it should do so, and he who drinks the smallest quantity on his journey through life will on the whole be the most healthy as well as the most virtuous and happy.

